# Mechanisms of thermal strengthening and deterioration in sandstone: Insights from characteristic stress and micro-crack analysis

**DOI:** 10.1371/journal.pone.0342561

**Published:** 2026-02-13

**Authors:** Lizhi Yang, Junjie Li, Daoxue Yang, Wen Chen, Mengjian Wang

**Affiliations:** 1 Hunan Polytechnic of Water Resources and Electric Power, Changsha, Hunan, China; 2 Hunan Engineering Research Center of Intelligent Inspection and Digital Maintenance for Hydraulic Engineering, Changsha, Hunan, China; 3 Zhejiang Design Institute of Water Conservancy & Hydroelectric Power Co., Ltd, Hangzhou, Zhejiang, China; 4 School of Civil Engineering and Surveying & Mapping Engineering, Jiangxi University of Science and Technology, Ganzhou, Jiangxi, China; 5 Zhejiang Academy of Surveying and Mapping, Hangzhou, Zhejiang, China; Universiti Malaysia Pahang Al-Sultan Abdullah, MALAYSIA

## Abstract

With the global push towards energy transition, understanding the mechanical behavior of rocks under high-temperature conditions is becoming increasingly significant, such as nuclear waste disposal. Previous studies have demonstrated that the high-temperature heating has a great impact on the physical and mechanical properties of sandstone, exhibiting both thermal strengthening and deterioration phenomena. However, research on the characteristic stress and microcrack evolution of sandstone under thermal strengthening and deterioration conditions remains limited. The objective of this study is to examine the characteristics of stress, microcrack evolution, and failure mechanisms in sandstone under a range of high temperatures. Sandstone samples were heated respectively to 200°C, 400°C, 600°C and 800°C. The deformation and failure processes were monitored using acoustic emission (AE) and digital image correlation (DIC) techniques. The evolution of characteristic stress and microcrack development under both thermal strengthening and deterioration conditions was analyzed. The results show that the crack closure stress and its threshold ratio are proportional to temperature. When sandstone exhibits strength deterioration, the crack initiation stress and its threshold ratio decrease with increasing temperature. Conversely, when strength enhancement is observed, the crack initiation stress increases, while the threshold ratio initially rises and then declines. Moreover, the effect of thermal treatment on axial deformation is more pronounced than on radial deformation, with the elastic constant and Poisson’s ratio gradually decreasing as temperature rises. Microcrack evolution was predominantly tensile across temperatures, but the macroscopic failure modes varied: monocline shear failure at 25°C, tensile failure at 200°C, 400°C, and 600°C, and a mixed shear-tensile failure at 800°C, where shear cracks became most prominent. This study enhances the understanding of sandstone’s mechanical behavior under high-temperature conditions and provides valuable insights for engineering applications involving thermal treatment.

## 1. Introduction

With the strategic goals of carbon peaking and carbon neutrality in place, the development of a clean and efficient energy technology system has become an important priority. Nuclear energy, as a low-carbon power source, poses a critical challenge regarding the safe disposal of nuclear waste. Compared to conventional above-ground storage methods, deep geological disposal offers long-term, isolated containment of high-level radioactive waste, significantly reducing potential risks to surface ecosystems and human health [1,2]. However, despite its recognized safety and sustainability benefits, deep geological disposal faces multiple challenges during project implementation and long-term operation [3,4]. The surrounding rock mass in a repository is subject to temperature changes due to decay heat, which may affect its mechanical properties and long-term stability, potentially leading to engineering hazards such as tunnel deformation, support structure failure, and fracture propagation in the surrounding rock [5,6]. Moreover, large geological faults and natural fracture systems could provide pathways for radionuclide migration, increasing the risk of transport into the biosphere and possibly causing groundwater contamination [7,8]. To ensure the long-term safety of the repository, it is essential to study the thermo-mechanical response characteristics and microfracture evolution mechanisms of the surrounding rock under multi-physics coupling conditions.

In recent decades, numerous studies have examined sandstone behavior under different temperatures [9–12]. The findings of research indicate that the physical property constant of rock mass typically declines under conditions of elevated temperature [13–15]. Simultaneously, micro-defects as microcracks and joints in the sandstone, develop to varying extents, leading to pore structure remodeling and the formation of secondary cracks [16]. These studies have explored the deterioration mechanisms under high temperatures of rock mechanical properties and micro-structures [17]. The disparity in the thermal expansion coefficients of rock components results in unequal expansion, which facilitates internal cracking. Additionally, the primary minerals found in sandstone are affected by thermal decomposition and phase transformation, which eventually exacerbates the deterioration of rock-bearing performance and leads to permanent structural collapse [18]. Nevertheless, as research progressed, numerous scholars discovered that the bearing capacity of sandstone augmented under specific temperature conditions when subjected to heating [19–22]. In this range, rock properties improve as the heating temperature increases, a phenomenon known as the thermal strengthening effect [23–25]. Zhang et al. [26] studied the mechanism of thermal strengthening and then proposed that the overall performance of sandstone is determined by the interplay between thermal damage and thermal strengthening. Within the thermal strengthening temperature range, the dehydration enhancement and healing of primary diagenetic minerals are vital factors, leading to improved mechanical properties of sandstone. However, most of the existing research focuses on the deterioration mechanisms of rock mechanical properties at high temperatures, with limited studies addressing the characteristic stress and microcrack evolution in sandstone under thermal strengthening conditions.

Currently, the effect of high temperature on the bearing capacity and structure of rocks has been studied by many researchers through heating experiments at different temperatures. These papers have identified thermal strengthening and deterioration phenomena depending on the treatment temperature. For instance, Zhu et al. [27] conducted static mechanical tests on sandstone heated at varying temperatures. It was found that the compressive limit stress of the sample initially rises and then falls as the heating temperature rises. Additionally, the values of the threshold ratios of crack initiation stress and damage stress all showed a gradual downward trend. Yu et al. [28] investigated the impact of cyclic thermal shock on Beishan granite from Gansu Province. It was revealed that increased thermal shock cycles significantly altered the crack distribution pattern of rock. Rathnaweera et al. [16] studied the effects of heating from room temperature to 1000°C and different temperature reduction steps on the mechanical behavior of sandstone. The results show that the compressive strength, elastic constant, cracking and failure stress of sandstone heated at 600°C will increase under any cooling mode, followed by a gradual decline after exceeding 600 °C.

Stress damage to rock is gradual, which includes the closure, propagation, aggregation, and eventual formation of macrocrack [29]. From the perspective of microcrack evolution, studying the patterns of crack initiation, propagation, and coalescence during the progressive failure of rock can provide a deeper understanding of its mechanical behaviors [30]. Additionally, the characteristic stress represents a significant variable in rock safety analysis [31]. The initial stress of crack is helpful to judge the pee strength of brittle rock, and the damage stress of crack is an indicator for evaluating the long-term strength of rock [32]. During loading, part of the strain energy stored in the rock is released, thus forming AE signals [30,33]. Therefore, it is necessary to understand the evolution of AE signal parameters during rock deformation and destruction, and the microcrack evolution under external load can be better understood [34–36]. Additionally, DIC can detect of crack initiation and propagation by analyzing digital speckle images during rock deformation. DIC technology allows for direct observation of the rock fracture evolution by examining the strain or displacement fields, and it helps interpret AE data obtained during testing [37–40].

In summary, most prior work has been devoted to high-temperature weakening of rocks and macroscopic strength changes, whereas the integrated investigation of characteristic stress evolution and coupled microcrack behavior (via AE and DIC) under both thermal strengthening and deterioration regimes has been lacking. In view of this, this article will conduct uniaxial compression tests on sandstone after heating at high temperatures of 25 °C, 200 °C, 400 °C, 600 °C, and 800 °C. The reason for choosing 800 °C as the highest temperature is because previous studies have shown that the mineral/chemical composition of sandstone changes at this temperature, causing the thermal strengthening effect of sandstone to shift towards degradation effect [25]. At the same time, the study utilizes AE and DIC testing techniques, in combination with the strain method of crack volume and Gaussian mixture model theory, to carry out relevant research.

## 2. Materials and test methods

### 2.1. Sample preparation

**Fig 1:**
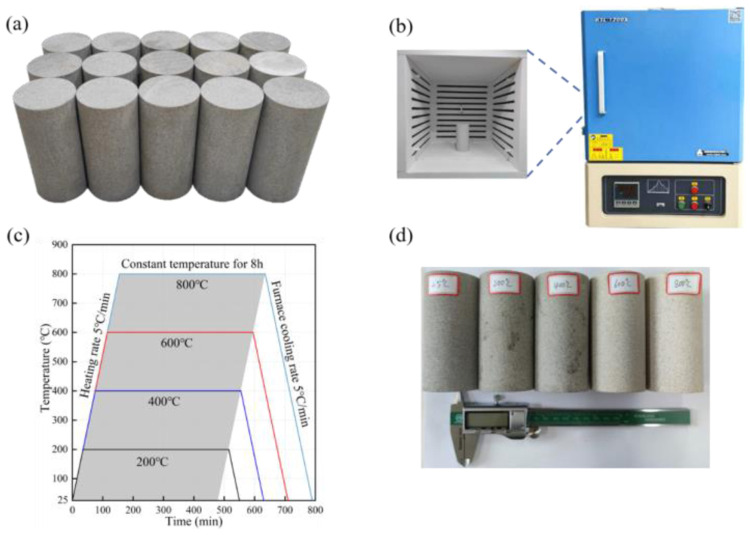
Sample and test method. **(a)** Sandstone sample before high temperature heating, **(b)** Box type resistance furnace, **(c)** High temperature heating scheme [26], **(d)** Sandstone samples after heat treatment.

The test material for this study is fine-grained sandstone which was gleaned from Hunan Province, China. As shown in Fig 1(a), the natural surface of the sandstone is yellowish-green. The texture is relatively uniform, with a low degree of weathering and no visible defects or cracks on the surface. All sandstone specimens were taken from the same fresh rock block. XRD indicates that Quartz (54.1%), Kaolinite (29.6%), illite (8.2%), Muscovite (6.7%), Pyrite (1.4%) are among the major mineral constituents [26]. The average density is approximately 2.16 g/cm³. By the specifications set forth for the indoor test, the rock samples necessary for the test are extracted from fresh test blocks with complete structure. Subsequently, according to the indoor testing standards of the International Society for Rock Mechanics (ISRM), the sample was cut into standard cylindrical samples (Φ50 mm × 100 mm). The flatness of both ends of the sample must be kept within 0.02 mm, and the perpendicularity between the end and the shaft to be kept below 0.25 °. All samples were polished to meet these criteria. Moreover, physical parameters can be quantified by means of a vernier caliper, including dimensions of the sample, mass, and longitudinal wave velocity. After that, samples with similar mass and wave velocity are selected, and they are used for subsequent tests.

### 2.2. High temperature heating scheme

In this study, a muffle furnace (KSL-1200X-M) (Fig 1(b)) is employed to heat sandstone samples at elevated temperatures. The experiment set up five groups of working conditions, with three samples in each group. Fig 1(a) shows the samples before heating. The experimental samples were heated respectively at 25 °C (room temperature), 200 °C, 400 °C, 600 °C, and 800 °C. T Randomly select a sample for uniaxial compression experiment at various set temperatures. Except for the room temperature group samples, all other groups of samples were placed in a heating device and subjected to high-temperature treatment at the set target temperature. During the heating process, temperature-induced cracks are created in the sample, and the heating rate causes them to initiate and expand. If the rate is too fast, thermal shock damage may occur, potentially causing macroscopic rupture during testing. [28] To avoid this, the sample is heated under the control of the equipment and reaches the setting temperature at the rate of 5 °C per minute. For the internal and external temperatures to reach a uniform state, the sample must be heated at the target temperature for a continuous period of 8 hours. Then, close the device and wait for the sample to cool naturally. Fig 1(c) is the heat treatment scheme. Additionally, the room temperature samples were dried to eliminate any free water inside that might affect the mechanical test results. The mass and wave velocity of the sandstones were remeasured after completing the heating.

In Fig 1(d), the samples remained intact after heat treatment, but their apparent color changed significantly with increasing temperature. At 400 °C, the color shifted from yellow-green to light green. And at 600 °C, it further changed to grayish-white. Under temperature heating, the average mass of samples is 510.18 g at room temperature, 508.86 g at 200°C, 507.30 g at 400°C, 490.88 g at 600°C, and 488.37 g at 800°C, respectively. From these results, it is easy to see that mass of the sample is seriously affected by high temperature.

### 2.3. Mechanical test equipment

**Fig 2:**
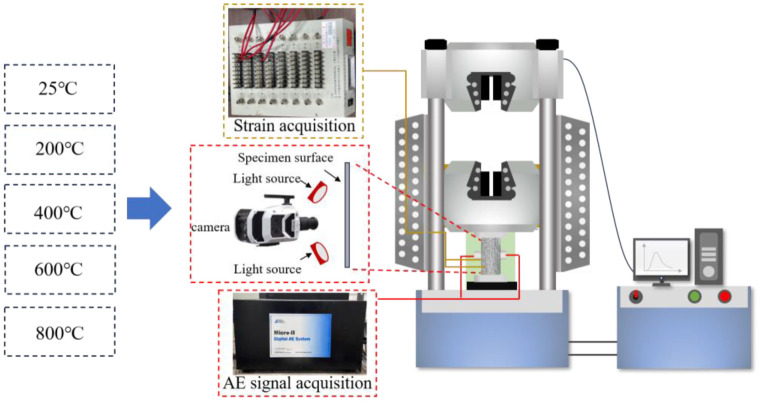
Schematic diagram of experimental equipment.

To carry out the examination of microcrack evolution and mechanical response characteristics of sandstone samples at high temperatures, the mechanical loading experiments of samples heated at different temperatures were carried out. During the tests, strain data and surface crack evolution information were collected simultaneously. Fig 2 shows the equipment used in the experiment.

A high-contrast random speckle pattern was applied to the specimen surface for DIC analysis. The sandstone surface was first painted white and then lightly sprayed with fine black mist to generate randomly distributed speckles with an average diameter of 0.5–1.0 mm. The imaging field of view encompassed the entire front face of the cylindrical specimen (50 mm in diameter). The camera captured an area of approximately 120 mm × 90 mm, providing a spatial resolution of ~0.03 mm/pixel. Each speckle thus covered roughly 10–30 pixels, ensuring a dense and well-resolved pattern for correlation.

A local subset-based DIC algorithm was employed, implemented using Vic-2D software. A subset size of 21 × 21 pixels and a step size of 5 pixels were adopted, with displacement matching based on a normalized cross-correlation criterion. Strain fields were derived from the displacement data using a central difference scheme to obtain Lagrangian strain components. The analysis was conducted in two dimensions on the specimen’s planar surface, assuming plane deformation. A local subset-based method was selected instead of a global finite-element-based DIC approach, as the local algorithm more accurately preserves sharp strain gradients characteristic of localized deformation zones.

For this study, the mechanical compression test was performed on a WAW-600B electro-hydraulic servo testing machine. The WAW-600B electro-hydraulic servo testing machine is equipped with a load cell of ±0.1% FS accuracy. During the compression tests, the data acquisition system recorded the load and displacement signals at a sampling frequency of 50 Hz to capture the mechanical response accurately. This equipment measures critical parameters such as tensile, compressive, and shear strength for both rock and metal materials. The rate is set to 0.2KN per second by force loading. The application of Vaseline can make a good coupling between the AE sensor and the sampled rock surface, and the Nano-30sensors were then securely wrapped with transparent tape around the middle height of the sample at mid-height. This setup helps prevent data loss in case one sensor detaches during testing. To reduce environmental noise interference, the AE sensor threshold is set to 35dB, 1MSP is the waveform sampling frequency, and 40dB is the gain of the preamplifier.

## 3 Experimental results and analysis

### 3.1 Distribution law of characteristic stress in sandstone under high temperature

**Fig 3:**
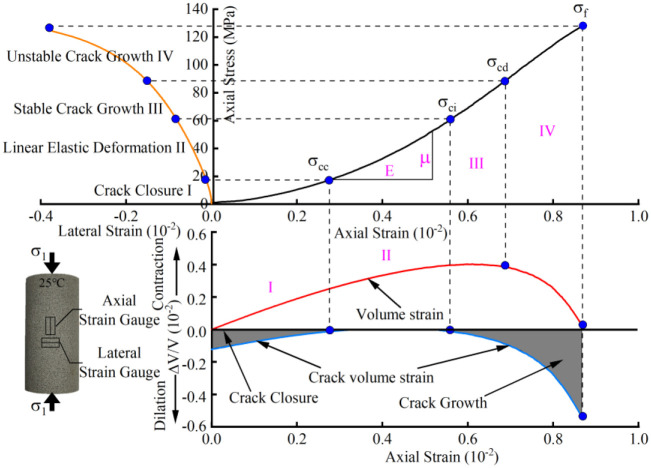
Characteristic stress calculation curve of sandstone under temperature.

According to previous studies, the failure process of rock can be composed of four deformation stages according to the characteristic stress [31]. This study on the closure stress of crack $\sigma_{cc}$ , initiation stress of crack $\sigma_{ci}$ and damage stress $\sigma_{cd}$ of the samples under high temperatures were determined by the crack volume strain method, and the lateral strain method verifies the calculation results. According to the above scheme, Fig 3 shows the division of each stage. Due to length limitations, only samples at room temperature are described in this study.

The volumetric strain of the rock specimen under uniaxial compression conditions can be expressed as follows:


$$\varepsilon_{V}=\frac{\Delta V}{V}=\varepsilon_{\text{1}}+\text{2}\varepsilon_{\text{2}}$$ (1)

Where, $\varepsilon_{V}$ denotes the volumetric strain. $\varepsilon_{\text{1}}$ denotes the axial strain. $\varepsilon_{\text{2}}$ denotes the lateral strain. The total strain of rock materials can be approximated as the sum of the elastic volumetric strain and the crack-induced volumetric strain. Therefore, the crack-induced volumetric strain of the rock specimen can be calculated using the following equation:


$$\varepsilon_{v}^{c}\text{=}\varepsilon_{V}-\varepsilon_{e}=\varepsilon_{V}-\frac{\text{1}-\text{2}\mu }{E}\sigma$$ (2)

Where, $\varepsilon_{v}^{c}$ denotes the crack-induced volumetric strain. σ denotes the axial stress.

In stage I (crack closure stage). Due to the gradual closure of microcracks in sandstone, the axial stress-strain curve is nonlinear. This state is transient until the stress on the sandstone reaches the stress level required for crack closure. In stage Ⅱ (linear elastic deformation stage), the deformation of sandstone is recoverable, and its stress and strain curves are in positive proportion. When the external load is unloaded, the volume strain of the sandstone crack returns to zero. [27] When the stress sustained by the sandstone structure reaches the stress required for crack initiation, the sandstone deformation enters stage III (stable crack propagation stage). At this stage, the microcracks in sandstone grow steadily, and the stress value and crack distribution position control its propagation behavior. In stage IV (unstable crack propagation stage), Micro-fractures in sandstone grow violently and form large fractures through expansion, condensation and nucleation. At this stage, the strain of sandstone is considerable, and the deformation mode shifts from axial contraction to lateral expansion. The initial stress at this stage is defined as damage stress. When the load of sandstone reaches the peak value, its volumetric strain rapidly decreases to close to zero. The sandstone forms macro fracture zone, which causes the instability of the sample.

In this study, four characteristic stresses and their threshold ratios of sandstone are statistically analyzed, and the results reveal the evolution law of characteristic stress of sandstone after heating.

**Fig 4:**
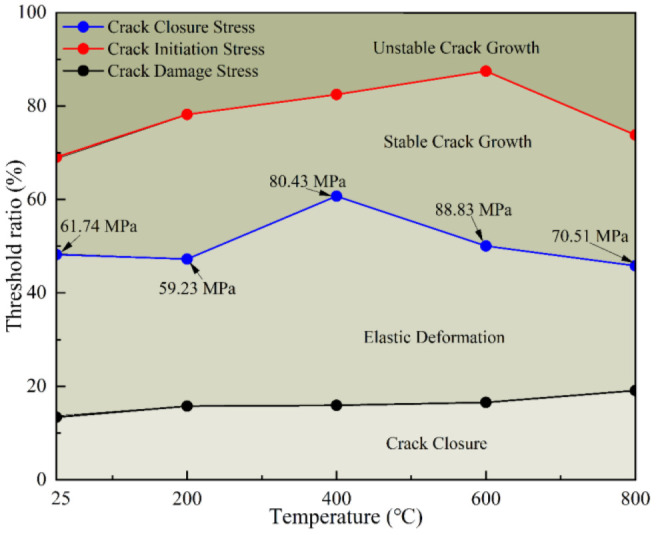
Characteristic stress threshold ratio curves after different temperature effects.

As shown in Table 1 and Fig 4. After heating, the thermal effect of temperature on the sample makes the strength of sandstone show obvious “strengthening” and “deterioration”. When sandstone is heated from room temperature to 200°C, the stress required for its failure decreases from 128.05 MPa to 125.33 MPa. Moreover, the stress required for failure decreases from 164.33 MPa to 153.89 MPa when the sample is heated from 600°C to 800°C. In these two temperature stages, the strength of sandstone samples showed a significant “deterioration effect”. The strength of samples increases from 125.33 MPa to 164.33 MPa, When the heating temperature is within the range of 200°C ~ 600°C, and displaying a significant thermal “strengthening effect”. From the perspective of the microstructure evolution characteristics and main mineral composition of samples after heating, the research team has studied the strength strengthening and strength deterioration mechanism of sandstone under different temperatures heating. For details, please refer to the literature 26. This study will not elaborate on mechanism of heat effect on strength strengthening and strength deterioration.

**Table 1:** Relevant parameters of sandstone samples after heating.

T (°C)	Characteristic stress (MPa)				Characteristic stress threshold ratio (%)			$E_{s}$ GPa	μ
	$\sigma_{cc}$	$\sigma_{ci}$	$\sigma_{cd}$	$\sigma_{f}$	$\sigma_{cc}/\sigma_{f}$	$\sigma_{ci}/\sigma_{f}$	$\sigma_{cd}/\sigma_{f}$		
25	17.13	61.74	88.43	128.05	13.38	48.21	69.06	22.80	0.26
25	16.87	60.34	87.94	127.56	13.23	47.30	68.94	21.94	0.25
25	17.65	62.31	89.57	128.93	13.69	48.33	69.47	23.32	0.26
200	19.74	59.23	97.99	125.33	15.75	47.26	78.19	22.26	0.25
200	19.03	58.72	98.41	124.89	15.24	47.02	78.79	22.78	0.25
200	19.98	59.36	98.13	125.64	15.90	47.25	78.10	21.98	0.26
400	21.08	80.43	109.22	132.43	15.92	60.73	82.47	22.71	0.24
400	22.03	79.51	110.25	133.62	16.49	59.50	82.51	22.84	0.23
400	21.37	80.96	108.17	131.99	16.19	61.34	81.95	22.95	0.24
600	27.19	88.83	143.74	164.33	16.55	50.06	87.47	20.50	0.17
600	26.48	90.24	142.36	163.28	16.22	55.28	87.18	20.94	0.17
600	27.39	89.27	141.67	160.49	17.06	55.26	88.27	19.57	0.18
800	29.38	70.51	113.57	153.89	19.09	45.82	73.80	14.79	0.13
800	30.84	71.23	112.94	152.98	20.16	46.56	73.83	14.24	0.13
800	28.13	68.32	111.37	150.34	18.71	45.44	74.71	14.96	0.12

From the experimental results, the stress required for crack closure increases with the temperature increasing, reaching a value of 29.38 MPa from an initial 17.13 MPa, this is like its threshold ratio, which increases from 13.38% to 19.09%. When the temperature was between 25°C and 200°C, and again between 600°C and 800°C, the sandstone exhibited a thermal deterioration effect, with both the crack initiation stress and its threshold ratio showing a gradual decline. When the temperature is between 200°C and 600°C, the sandstone demonstrated a thermal strengthening effect, with crack initiation stress gradually increasing, and its stress threshold ratio increases first and then decreases. Under heating at various temperatures, the crack damage stress and its threshold ratio followed a pattern of initial increase, followed by a decrease. The results of characteristic stress analysis show that the number of hot cracks increases with the increase of heating temperature, resulting in the gradual increase of crack closure stress and threshold ratio. The competing influences of thermal crack propagation (which causes deterioration) and sandstone dehydration/dehydroxylation (which causes strengthening) significantly affect the characteristic stress response of the sandstone samples.

The corresponding increase in the threshold ratio ($\frac{\sigma_{cc}}{\sigma_{f}}$) from 13.4% to 19.1% implies that nearly one-fifth of the total failure stress is consumed during the crack closure stage at 800 °C. This phenomenon suggests the presence of microcrack interlocking, where cracks cannot be easily closed under low stress due to frictional resistance along rough surfaces or partial mineral healing that enhances their mechanical interlock. Once these cracks are forcibly closed, the sandstone exhibits partial stiffness recovery, allowing it to sustain additional load before crack initiation and eventual failure.

### 3.2 Basic laws of deformation characteristics of sandstone under high temperature

**Fig 5:**
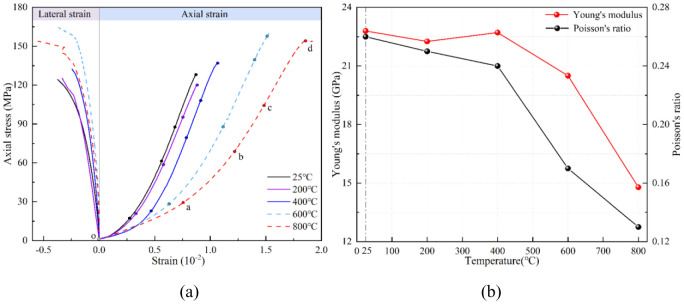
Show sandstone samples heated at different temperatures. **(a)** Stress-strain, **(b)** Mechanical parameters.

During the loading process of the sample, the expansion of the crack surface parallel to the loading direction causes its lateral deformation, and the closure of the crack surface perpendicular to the loading direction causes its axial deformation. The joint analysis of the mechanical curve of sandstone is helpful to explore the deformation behavior of the sample under different heating temperatures. Fig. 5 shows the entire stress-strain curve of sandstone samples after heating and the elastic modulus and Poisson’s ratio at corresponding temperatures.

The four deformation stages of the heated sandstone sample correspond to stage I(o-a), stage II(a-b), stage III(b-c) and stage IV(c-d) on the curves, which can be seen from Fig 5(a). After heating, the sandstone sample has axial compression deformation and radial expansion deformation. At the crack closure stage, the temperature has little effect on the radial deformation of the sample, and the stress-strain curves are basically consistent. When the bearing stress of the heated sample reaches the crack closure stress, the difference between the stress radial strain curves at 25°C and 200°C is relatively small, and the same is true between 400°C and 600°C. However, compared with other temperatures, the curve at 800°C is quite different. Compared with the radial, the axial curve is more affected by temperature. Among them, the difference between the curves of axial at 25°C and 200°C is relatively tiny. When the temperature reaches 200°C, the strain of axial corresponding to the four deformation stages of the sample also increases as the temperature increases. In addition, in Fig 5(b), the average elastic modulus of the sample decreases continuously as the heating temperature increases, and the average Poisson’s ratio also follows the same pattern of change.

The sandstone consists primarily of quartz (~54%) and a considerable proportion of clay minerals (kaolinite and illite, ~ 38% combined, according to XRD results). Quartz undergoes a well-known α-β phase transition near 573 °C, accompanied by a sudden volume increase and a generally higher thermal expansion coefficient than that of clays. In contrast, clay minerals typically shrink upon dehydration (loss of interlayer water) and exhibit anisotropic expansion, expanding preferentially along certain crystallographic axes. The resulting mismatch in expansion between quartz grains and the surrounding clay matrix induces microcracks, mainly oriented perpendicular to grain boundaries. These thermally generated cracks preferentially reduce stiffness.

In summary, the analyses indicate that the resistance of sandstone to overall deformation under high temperature heating is reduced. Compared to radial deformation, the deformation in the direction of sandstone force loading is more significantly affected by temperature.

### 3.3 Progressive failure process of sandstone under high temperature

**Fig 6:**
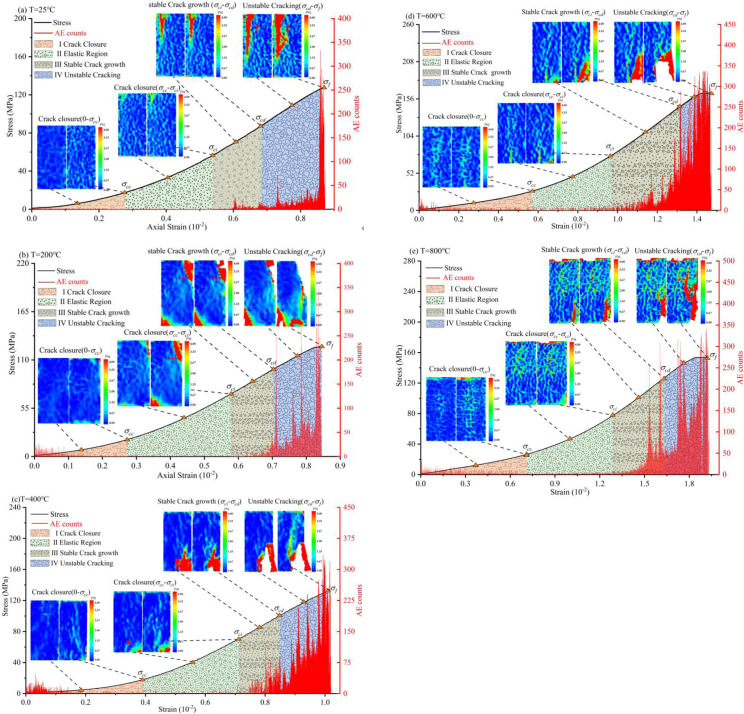
Continued.

To explore the progressive failure process of sandstone after heating at varying temperatures, the DIC method was used to analyze the principal strain field of the samples. As shown in Fig 6, due to space limitation, only the principal strain field images of each characteristic stress point and its middle-stress level are displayed in this paper. Under the action of external load, a part of the strain energy will be stored in the rock. The evolution of microcracks and surface principal strain fields in rocks is intrinsically related to AE. Fig 6 shows the statistics of AE activity in sandstone. In addition, the color-code on the right side of the strain nephogram indicates the variation range of the principal strain. At the same time, the color code area is divided into low strain area (blue), medium strain area (green) and high strain area (red) to better describe the change law of the principal strain field.

The principal strain field nephogram of sandstone at each deformation stage is shown in Fig 6. The surface principal strain field is mainly distributed in the low and medium strain region and less in the high-strain region when the sample is in the initial fracture closure stage. The proportion of low-strain regions declines gradually with the increase in heating temperature, while the proportion of medium-strain regions is the opposite. The AE activity intensity of the sample heated at temperature is stronger. In the crack closure stage, the AE activity is mainly related to the friction activity strength of the microcrack interface in the sample under load. The above shows that temperature heating increases the nonuniformity and microcrack density of samples, while the intensity of AE activity is not correlated positively with temperature. The higher the heating temperature, the greater the connectivity and size of the thermal cracks in the sample, which hinders the propagation of AE signals. Therefore, the AE activity frequency of samples heated at 800°C is lower than that at 400°C.

Stage I: Crack Closure-Predominantly low-amplitude “frictional” signals are recorded, arising from crack surface rubbing and closure. The AE event rate remains low, representing mostly background activity, as no new cracks are yet formed. The strain field appears uniformly blue (low strain) across the sample, with only minor, isolated medium-strain patches reflecting uneven crack closure. As noted in the revised text, AE activity at this stage mainly results from interfacial friction and does not vary significantly with temperature, as the deformation is largely recoverable and produces weak AE signals.

Stage II: Linear Elastic Deformation- Very few AE events occur in this stage, and those recorded exhibit low amplitudes, reflecting fully recoverable deformation without crack initiation. The strain field remains mostly uniform, showing a slight overall increase in strain but no distinct localizations or “hot spots.” AE activity was minimal, and DIC images displayed predominantly blue and green zones, confirming uniform elastic deformation.

Stage III: Stable Crack Growth- The AE event rate rises sharply, characterized by numerous medium-amplitude events and evolving frequency content. Tensile-type AE signals, typically with short rise times and higher frequencies, dominate as microcracks nucleate and propagate steadily. The cumulative AE count increases significantly, often reaching hundreds of kilohertz. Correspondingly, localized high-strain regions (red zones) emerge in the strain field at crack initiation sites. With increasing temperature, these localizations become more irregular and intense, reflecting the influence of thermal heterogeneity. In the revised text, we note that “the proportion of high-strain regions shows a gradual increase, and AE activities become more intense than in the previous stages.” The formation of multiple red patches coincides with frequent AE bursts, confirming that microcrack propagation is the main AE source during this stage, even under thermal strengthening conditions.

Stage IV: Unstable Crack Growth- More high-amplitude AE events typically occur near or at peak stress, corresponding to through-going fracture formation. These signals often exhibit longer rise times and lower frequencies, characteristic of shear or mixed-mode cracking, though sudden tensile ruptures can produce extremely high amplitudes. AE energy spikes markedly as the rock approaches ultimate failure. At and beyond *σ*_cd_, the DIC strain field shows intense localization, with contiguous red zones forming and expanding rapidly to delineate the macrocrack path. At failure, DIC images capture a coalesced high-strain band, often accompanied by visible surface spalling or fracture propagation.

### 3.4 Evolution characteristics of microcracks during progressive failure of sandstone under high temperature

The mechanisms of microcrack propagation in progressive rock failure are various. It can be divided into tensile microcracks and shear microcracks [33,35,41]. The characteristics of AE signals associated with these two kinds of microcracks also have significant differences in the process of incubation and propagation. The AE signals associated with shear microcracks have the characteristics of short rise time and relatively large amplitude, while tensile microcracks are on the contrary [34,42]. Therefore, the types of microcracks can be distinguished by analyzing the differences in AE parameters. The RA and AF values of AE were statistically analyzed to obtain the distribution characteristics of AE rise time, duration, ring count and amplitude. Calculate the RA and AF values of AE from the formula (3,4).


$$\text{AF =}\frac{\text{Count}}{\text{Duration}}\text{(kHz)}$$ (3)

$$\text{RA =}\frac{\text{Rise time}}{\text{Amplitude}}\text{(ms/V)}$$ (4)

**Fig 7:**
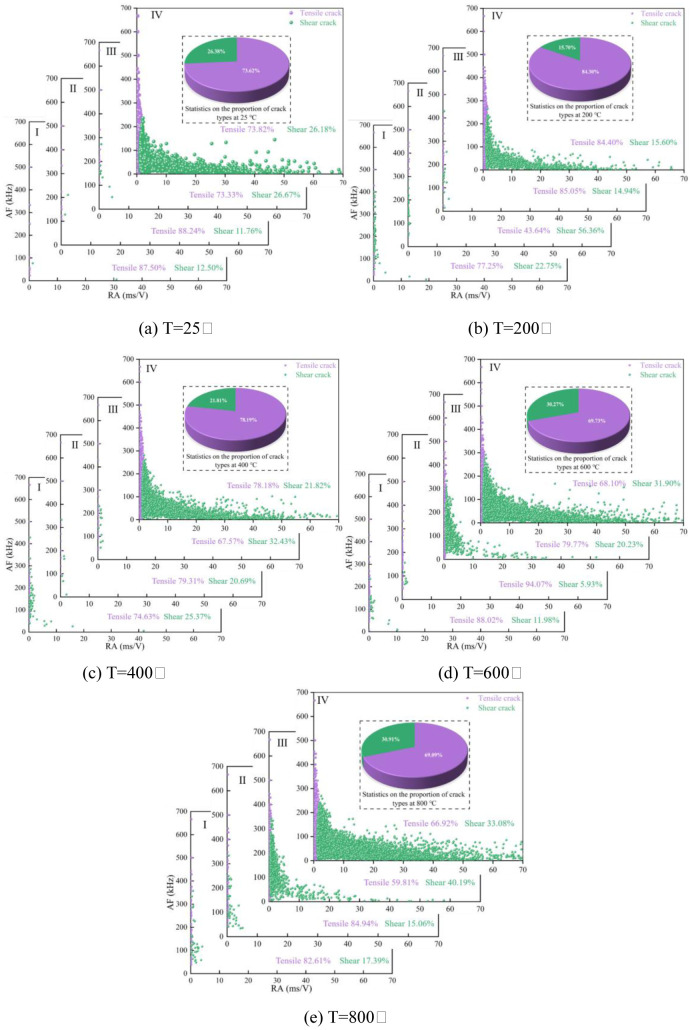
Changes of RA-AF during uniaxial compression of sandstone under different temperatures.

To explore the evolution of microcracks in heated sandstone samples during progressive failure, this paper uses a model of Gaussian mixture to classify the RA and AF values of AE generated during progressive failure, and then identifies shear and tensile cracks. Due to the limitation of space, this paper will not repeat the Gaussian mixture model. See resources for details [43]. To better achieve the study purpose, the ratio of tensile and shear crack in four deformation stages is statistically analyzed using RA and AF values. Fig 7 and Table 2 show the experimental results.

**Table 2:** Evolution of microcracks in sandstone samples at different stress stages under high temperature.

T (°C)	Stage Ⅰ (%)		Stage Ⅱ (%)		Stage Ⅲ (%)		Stage Ⅳ (%)		Total Process (%)	
	Tensile	Shear	Tensile	Shear	Tensile	Shear	Tensile	Shear	Tensile	Shear
25	87.50	12.50	88.24	11.76	73.33	26.67	73.82	26.18	73.62	26.38
200	77.25	22.75	43.64	56.36	85.05	14.94	84.40	15.60	84.30	15.70
400	74.63	25.37	79.31	20.69	67.57	32.43	78.18	21.82	78.19	21.81
600	88.02	11.98	94.07	5.93	64.77	35.23	68.10	31.90	69.73	30.27
800	82.61	17.39	84.94	15.06	59.81	40.19	66.92	33.08	69.09	30.91

It can be seen from Fig 7 that range 0 ~ 700kHz is the distribution region of tensile crack AF value, with 0 ~ 500kHz representing its main concentration range. Moreover, 0 ~ 1ms/V is the leading distribution range of its RA value. The shear microcrack AF distribution range is 0 ~ 300kHz, while the concentration region of the RA value is 0–40ms/V. In the process of progressive failure of sandstone, the distribution of shear microcracks RA-AF value is significantly affected by heating temperature. At room temperature, the primary distribution range of shear microcracks AF is 0 ~ 100kHz; at 600°C, this range shifts to 0 ~ 200kHz; at 800°C, it extends to 0 ~ 300kHz. However, the RA value of shear microcracks is mainly concentrated in the region that is affected relatively less by temperature. The distribution of shear microcracks becomes denser with the increase of temperature in the region of 0 ~ 40ms/V.

As can be observed in Fig 7 and Table 2, AE signal strength is relatively low at the stage of crack closure and elastic linear deformation. In the stable crack growth stage, the associated AE activity also gradually increased in the slow failure process with the initiation and propagation of microcracks in the sandstone. The dominant type of microcracks at this stage is tensile microcracks. With the increase in heating temperature, the proportion of tensile microcracks gradually decreases while the proportion of shear microcracks continues to increase. The AE activity of the sample is the strongest in the unstable crack growth stage. When heated at 25°C ~  200°C, the proportion of tensile microcracks increases gradually with temperature increasing, but the proportion of tensile microcracks changes in the opposite direction when heated at 200°C ~  800°C.

## 4. Discussion

### 4.1. Effect of thermal cracks on evolution of principal strain field in progressive failure process of sandstone

There is a close correlation between the surface principal strain field and the thermal crack distribution of sandstone samples under thermal deterioration, which has been confirmed by previous studies. However, there are few reports on the thermal strengthening effect of sandstone [38]. To explore the relationship between the distribution of thermal cracks in sandstone and the evolution of its surface principal strain field under thermal strengthening. SEM technology was used to detect the distribution characteristics of thermal cracks in samples after heating. With reference to the control group (25°C), the internal thermal crack density and distribution of sandstone samples heated at 200°C and 400°C are relatively small. Therefore, this paper only gives the microscopic image in the samples heated at room temperature, 600°C and 800°C, and the principal strain nephogram information corresponding to the positions of each characteristic stress point is displayed.

**Fig 8:**
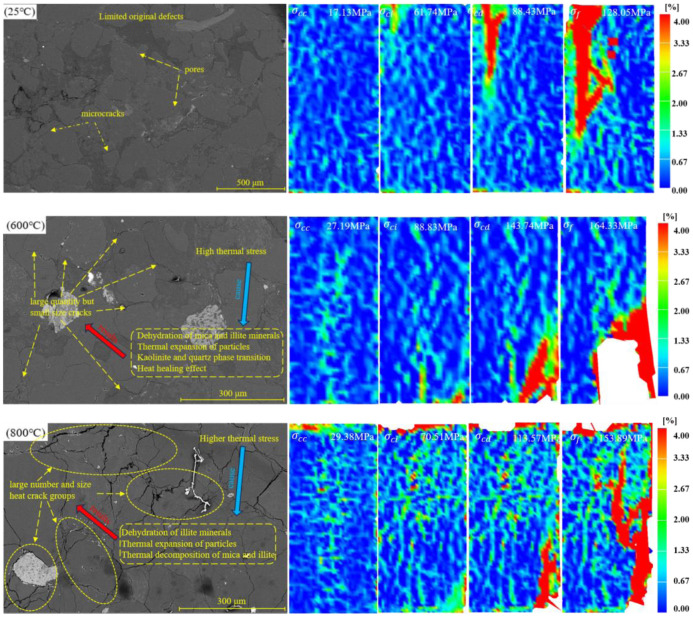
SEM images [26] and DIC main strain field nephogram of sandstone samples after heating at different temperatures.

As shown in Fig 8, at room temperature, the internal structure of sandstone is dense, and there are relatively few primary microcracks. The sandstone sample has good cementation between the internal particles and the cement at this temperature. The sandstone sample is undergoing deformation and failure, and its surface non-uniform principal strain shows gradual evolution. When the sandstone is loaded to the crack initiation stress, the internal microcracks expand in the left region of the upper-end section. When the sandstone is loaded to the damage stress, the microcracks in the left area of the upper end enter the stage of through evolution. When the load stress reaches the ultimate bearing stress of sandstone, the crack evolves and nucleates, forming a macrocrack zone, resulting in the failure mode dominated by tensile failure. When heated to 600°C, thermal cracks in samples will increase significantly, and a few cracks are connected. Thermal cracks are mainly distributed at the contact between particles and cement, and there are almost no thermal cracks penetrate cement or particles. At the same time, minerals such as mica and illite undergo dehydration and dehydroxylation reactions under high temperatures. This will lead to irrecoverable deformation of mineral particles and cement that make up the rock, and enhance the mechanical properties of mineral components such as mica and illite. Under the joint action of thermal deterioration and thermal strengthening, the mechanical properties of sandstone samples are significantly improved, which can be confirmed in Fig 5. The above test results show that the thermal strengthening effect plays a dominant role in the heating process 600°C. The thermal crack of samples expands rapidly, which significantly improves the heterogeneity. When sandstone is loaded to the stress required for crack closure, the proportion of the medium-strain zone in its main strain field increases significantly. However, When the load of sandstone does not reach the peak stress, there is no debris spalling on its surface. When the temperature is heated to 800°C, mica, kaolinite and other sandstone minerals undergo a thermal decomposition reaction, Causing a sharp increase in the number of thermal cracks. Most of the thermal cracks in sandstone are open at this temperature. The connectivity of thermal cracks is significantly enhanced, and crack groups are formed. When sandstone is loaded to damage stress, the surface of the sample appears to be a spalling phenomenon.

Although a lower heating rate is used, the thermal shock still effects the sample during the heating and cooling. Thermal shock damage causes relatively more microcracks on the edge of samples, while the internal thermal crack density is relatively low [15]. Therefore, the sandstone sample after heat treatment at 600°C has a significant stress concentration under the action of load, and the sample at 800°C has a surface fragment spalling phenomenon. This is because the surface structure of the sample is subjected to tensile stresses during the heating, while its internal structure is subjected to compressive stresses [44]. This causes the thermal stress to be higher than the bearing limit of the sample, and then forms a thermal damage region on the surface of the sample.

According to Griffith’s criterion, the crack extension behavior is not only related to its required surface energy but also to the crack density. The crack will expand only when the crack density exceeds the limit value [28]. Fig 8 shows that the internal thermal crack density of the sample is gradually increasing with the increase in heating temperature. Secondary cracks will appear in sandstone samples when they reach the necessary stress for crack initiation, which will lead to an increase in microcrack density. When the load stress is large enough, the microcrack density in the sample reaches a critical value, and the crack begins to expand. This will make the high strain ratio on the surface of the sample larger, accompanied by high-frequency AE fluctuations.

The progressive increase in the complexity and extent of high-strain zones with temperature visually indicates that the crack density is approaching a critical threshold, beyond which individual cracks begin to interact and coalesce. This interpretation is further supported by the observation that, at failure, the 800 °C specimen exhibited surface spalling and a through-going macrocrack, clear evidence that a critical damage state had been reached.

### 4.2. Effect of thermal cracks on AE activity and micro-fracture evolution mechanism

In Section 3.4, the ratio of two kinds of microcracks in sandstone samples was statistically analyzed. The findings presented in this paper are comparable to those reported by Zhu et al. [27]. They used CT technology to scan the rock sample and obtained the following conclusions: (1) The layout of shear-type cracks is strongly related to some factors, such as the location, angle and load of cracks. (2) Due to heating, the internal structure of sandstone is degraded to a high degree. Under the condition that the heating temperature of sandstone keeps rising, the crack distribution presents an apparent shear continuous plane, which makes the sandstone easier to diffuse along the internal defects and form shear failure under axial load.

**Fig 9:**
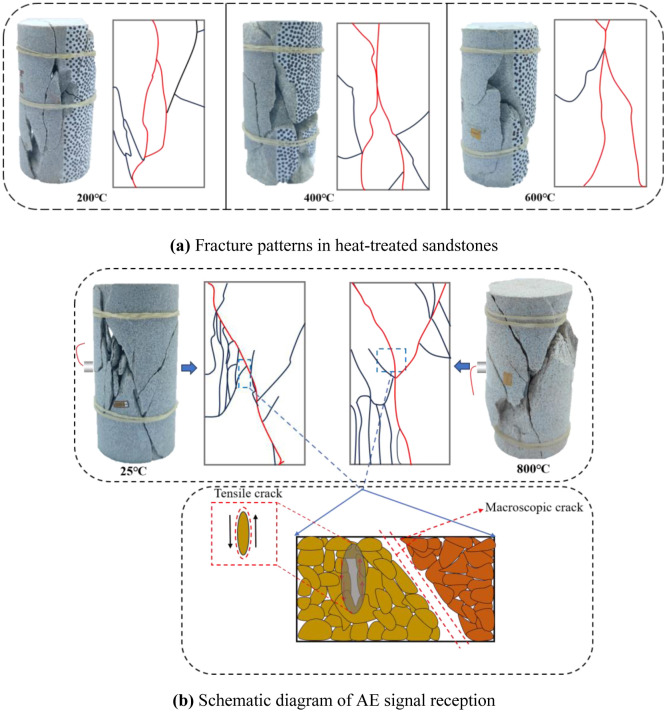
Schematic diagram of acoustic emission signal reception of shear microcracks [34].

This can be seen in Fig 7 and Table 2. Tensile crack is the primary type of microcrack in sandstone samples at various temperatures. However, there are some differences between the final fracture mode and the type of internal microcracks. As shown in Fig 9(a), when heated at 200°C, 400°C and 600°C, the microcracks in sandstone samples are mainly tensile. Accounting for 84.30%, 78.19% and 69.73% of the total microcracks, respectively. When sandstone fails, it shows tension fracture. The surface of the sample forms a fracture zone through the top and bottom surfaces, which is roughly parallel to the axial direction of the sample. At the same time, there are obvious transverse cracks around the two main cracks (red lines). When the temperature is 25°C and 800°C, The total proportion of tensile cracks in sandstone samples is 73.62% and 69.09%, respectively. However, the sandstone sample forms an independent shear fracture zone in macro failure, which is more like the formation of a shear fracture. Combined with the different propagation characteristics of AE signal in rock medium and air, the reason for this difference is speculated. Such a result may be affected by the position where the AE receiving sensor is pasted on the surface of the test piece, thus affecting the sensor’s regular reception of the shear crack signal by. This affects the reception of the shear signal by the AE receiving sensor [45]. As shown in Fig 9(b), under room temperature and 800°C, the dense areas of tensile and shear cracks of sandstone samples are roughly located on both sides of the main fracture zone. The tension crack signal is acquired first when the AE receiver sensor is affixed to the adjacent tension crack-intensive area. However, the propagation of the AE signal of the shear crack at the far end is hindered by the macro fracture zone, resulting in the phenomenon that the signal becomes weak or even lower than the threshold value, which affects the typical acquisition of acoustic signal by the sensor. Therefore, the difference between the dominant crack and failure mode of heated sandstone at 25°C and 800°C appears.

The results show that at room temperature and moderate heating (200–600 °C), most AE-detected microcracks were classified as tensile cracks. In contrast, at 800 °C, a markedly higher proportion of shear cracks was observed, approximately 30%. Correspondingly, the macroscopic failure mode transitioned from predominantly tensile splitting at 200–600 °C to mixed tensile–shear failure at 800 °C.

This transition is attributed to temperature-induced alterations in the clay matrix and grain boundaries. Below approximately 600 °C, the clay minerals (kaolinite and illite) retain partial cohesion and stiffness, despite dehydration. They remain brittle and even exhibit temporary strengthening, promoting crack propagation through or across grains in a tensile manner. Because the matrix still bonds the grains tightly, failure primarily occurs by tensile splitting once the rock’s tensile strength is exceeded, consistent with predominately tensile AE signatures.

Above 600 °C, however, the clay minerals begin to lose their binding capacity. Kaolinite undergoes dehydroxylation by 400–600 °C and transforms into amorphous metakaolin or related phases by 800 °C, while illite and muscovite begin to decompose or soften, possibly forming partially glassy, weakly cohesive phases. This process termed matrix plasticization significantly reduces the mechanical integrity of the clay cement. Concurrently, grain-boundary decohesion occurs as the interfaces between quartz grains and the clay matrix weaken. SEM observations at 800 °C reveal extensive intergranular cracking, confirming this degradation.

In the thermal deterioration regime (>600 °C), a significant portion of the applied work is dissipated through the creation of new crack surfaces and frictional sliding along them, rather than being stored elastically to sustain load. Consequently, the peak strength decreases. The energy that would otherwise be stored as elastic strain energy is instead consumed by damage-related processes. In other words, energy that could contribute to higher stress capacity is diverted into the formation of new fracture surfaces and the motion of rock fragments [45].

Up to approximately 600 °C, the rock can store more elastic energy, thereby maintaining or even enhancing its strength, as fewer new cracks are generated and some energy may contribute to structural tightening. Beyond 600 °C, however, each increment of strain produces numerous microcracks, as confirmed by SEM observations showing a continuous increase in crack density. The formation and sliding of these cracks consume energy through fracture and frictional dissipation, which does not contribute to load resistance. As a result, the rock’s load-bearing capacity diminishes even as the total input energy continues to increase.

### 4.3 Thermodynamic effect

The thermal strengthening and deterioration of sandstone are consistent with previously established thermal stress damage models for igneous rocks such as granite and basalt. Similar to granite, sandstone exhibits a thermal strengthening effect between 200 °C and 600 °C, which can be attributed to the dehydration and recrystallization of clay minerals, as well as partial healing of pre-existing cracks at moderate temperatures [46,22]. This mechanism reduces the effective porosity and enhances interparticle bonding, thereby improving the material’s mechanical properties. However, beyond 600 °C, the progressive decomposition of mica and kaolinite and the severe mismatch in thermal expansion coefficients among minerals promote intergranular cracking and lead to significant strength deterioration. This trend parallels the behavior observed in granite and basalt, where high-temperature exposure induces thermal stress concentration and crack coalescence [38,18].

In addition, the gradual increase in crack closure stress with temperature observed in this study is comparable to findings by Zhu [27] and Wang [41], who attributed the increase to thermal stress-induced microstructural reorganization and compaction of closed cracks at intermediate temperatures. Nevertheless, our SEM analysis (Fig 8) revealed that when the temperature reaches 800 °C, the number of open cracks increases sharply, and intergranular cracks become dominant, indicating severe grain boundary disruption and the formation of connected crack networks. This structural transformation corresponds well with observations in high-temperature granite, where extensive microcrack networks facilitate failure under lower incremental stress [18]. These similarities suggest that while the underlying mechanisms of thermal damage in sandstone share common features with granite and basalt, mineralogical differences (such as clay content) strongly influence the temperature range and magnitude of strengthening and weakening effects.

The results provide useful guidance for preliminary design and safety assessments in high-temperature rock engineering projects, such as geothermal energy extraction, underground nuclear waste repositories, and tunnel fire scenarios. The observed increase in sandstone strength up to approximately 600 °C suggests that short-term bearing capacity may not necessarily decrease at moderate temperatures and may even improve slightly. However, when temperatures exceed about 600 °C, a rapid reduction in strength occurs.

From an engineering perspective, these findings imply that prolonged exposure of sandstone structures to temperatures above 600 °C should be avoided where possible. If such exposure is unavoidable, a significant reduction in rock strength should be assumed. In the present study, a strength loss of approximately 10–20% was observed at 800 °C. In addition, the continuous decrease in elastic modulus with increasing temperature indicates that larger deformations in rock masses and support systems should be expected, even when compressive strength has not yet declined.

Furthermore, the observed evolution of microcracking mechanisms provides insight into potential failure modes under thermal loading. Tensile-dominated cracking may lead to spalling, whereas shear-dominated cracking may result in sliding-type failures. These distinctions are directly relevant to support design. Tensile spalling may require reinforcement such as rock bolts, while shear-related failures may necessitate strapping or confinement measures.

## 5. Conclusion

This study investigated the characteristic stress evolution and microcrack propagation of sandstone under thermal strengthening and deterioration conditions using AE and DIC techniques. The main conclusions are:

### Characteristic stress

Crack closure stress increased from 17.13 MPa (25 °C) to 29.38 MPa (800 °C), while the threshold ratio rose from 13.38% to 19.09%. Crack initiation stress exhibited a strengthening trend between 200 °C (59.23 MPa) and 600 °C (88.83 MPa), but decreased to 70.51 MPa at 800 °C.

### Deformation behavior

The elastic modulus decreased by 40% (from 22.80 GPa at 25 °C to 14.79 GPa at 800 °C), and Poisson’s ratio declined from 0.26 to 0.13, indicating higher compressibility and reduced stiffness at elevated temperatures.

### Microcrack evolution and failure mode

Tensile microcracks dominated (>69%) at all temperatures, but their proportion declined with temperature, while shear cracks increased to 30.19% at 800 °C, leading to a mixed tensile-shear failure mode at this extreme condition.

### Highlights

(1) The evolution law of characteristic stress of sandstone under the condition of thermal strengthening and deterioration is analyzed.
(2) The relationship between the characteristic stress and the evolution characteristics of microcracks is discussed qualitatively.
(3) The evolution and failure mechanism of sandstone microcracks are revealed under different thermal effects.
(4) The understanding of mechanical behavior of sandstone under high temperature is deepened.

## Supporting information

S1 File Data. (ZIP)
